# SCG2 is a Prognostic Biomarker Associated With Immune Infiltration and Macrophage Polarization in Colorectal Cancer

**DOI:** 10.3389/fcell.2021.795133

**Published:** 2022-01-03

**Authors:** Hao Wang, Jinwen Yin, Yuntian Hong, Anli Ren, Haizhou Wang, Mengting Li, Qiu Zhao, Congqing Jiang, Lan Liu

**Affiliations:** ^1^ Department of Oncology, Shanghai East Hospital, Tongji University School of Medicine, Shanghai, China; ^2^ Hubei Clinical Center and Key Lab of Intestinal and Colorectal Diseases, Wuhan, China; ^3^ Department of Gastroenterology, Zhongnan Hospital of Wuhan University, Wuhan, China; ^4^ Department of Colorectal and Anal Surgery, Zhongnan Hospital of Wuhan University, Wuhan, China

**Keywords:** colorectal cancer, SCG2, prognosis, tumor immunology, macrophage polarization

## Abstract

Colorectal cancer (CRC) is the second most lethal malignancy around the world. Limited efficacy of immunotherapy creates an urgent need for development of novel treatment targets. Secretogranin II (SCG2) is a member of the chromogranin family of acidic secretory proteins, has a role in tumor microenvironment (TME) of lung adenocarcinoma and bladder cancer. Besides, SCG2 is a stroma-related gene in CRC, its potential function in regulating tumor immune infiltration of CRC needs to be fully elucidated. In this study, we used western blot, real-time PCR, immunofluorescence and public databases to evaluate SCG2 expression levels and distribution. Survival analysis and functional enrichment analysis were performed. We examined TME and tumor infiltrating immune cells using ESTIMATE and CIBERSORT algorithm. The results showed that SCG2 expression was significantly decreased in CRC tumor tissues, and differentially distributed between tumor and adjacent normal tissues. SCG2 was an independent prognostic predictor in CRC. High expression of SCG2 correlated with poor survival and advanced clinical stage in CRC patients. SCG2 might regulate multiple tumor- and immune-related pathways in CRC, influence tumor immunity by regulating infiltration of immune cells and macrophage polarization in CRC.

## Introduction

Colorectal cancer (CRC) is the fourth most commonly diagnosed cancer, causing more than ninety thousand deaths worldwide every year (Bray et al., 2018; Siegel et al., 2020). Although diagnosis and treatment have been improved substantially, the prognosis of patients remains poor, especially in those with higher TNM stage (Luebeck et al., 2013; Miller et al., 20162016). Abnormal expression of multiple genes is usually associated with the occurrence of CRC (Meyerson et al., 2010). However, the molecular mechanisms underlying CRC remain unclear. It is urgent to identify novel diagnostic biomarkers and prognostic predictors for this disease.

New treatment modalities have been proposed for CRC, such as immunotherapy (Overman et al., 2018). Recently, studies have shown that the tumor microenvironment (TME) significantly affects CRC progression and therapeutic efficacy (Halama et al., 2011). Monoclonal antibodies against programmed cell death 1 (PD-1), programmed death ligand-1 (PD-L1) and cytotoxic T-lymphocyte–associated antigen 4 (CTLA4) have been proven effective in clinical trials (Rotte, 2019). However, anti-PD-1/PD-L1 therapy is unsatisfactory in metastatic CRC (Le et al., 2015), whereas anti-CTLA-4 treatment showed a limited response rate in advanced CRC (Chung et al., 2010). Therefore, it is of paramount importance to find novel immunotherapy targets of CRC.

Secretogranin II (SCG2) is a member of the tyrosine-sulphated granin family expressed in endocrine, neuroendocrine and neuronal tissues (Troger et al., 2017). It is relevant to secretory vesicle formation and packaging peptide hormones into vesicles (Beuret et al., 2004). SCG2 has an important role in enhancing endothelial cell proliferation, migration, and angiogenesis (Albrecht-Schgoer et al., 2012; Hannon et al., 2018). Studies have revealed that the derived peptides of SCG2, such as secretoneurin (SN) and EM66, are useful markers of neuroendocrine tumors (Guillemot et al., 2012). Besides, SCG2 could predict prognosis in Non-small cell lung cancer as a potentially secreted biomarker (Cury et al., 2019), reflect the TME of lung adenocarcinoma (Wang and Chen, 2020) and bladder cancer (Luo et al., 2020). Recently, Liu et al. (2020) identified SCG2 was a stroma-related gene and predicted poor outcomes in CRC patients. However, the relationship between SCG2 and tumor immunity of CRC is largely unknown, and the mechanisms underlying it remain to be intensively investigated.

This study analyzed SCG2 expression profiles and described its potential prognostic value, multiple biological functions, related signal pathways in CRC. This report’s findings revealed the potential role SCG2 played in regulating tumor immunity and provided a possible mechanism based on bioinformatic analysis.

## Materials and Methods

### Specimen Collection

We obtained 61 pairs of CRC primary tumor tissues and adjacent tissues from patients who underwent surgery at Zhongnan Hospital of Wuhan University (Wuhan, China) between June 2019 and September 2019. None of the patients received any preoperative therapy such as adjuvant chemotherapy or radiotherapy. Samples of the collected tissues were preserved in RNAlater Stabilization solution (Invitrogen, United States) and stored in the department of Biological Repositories (Zhongnan Hospital of Wuhan University). Our study was approved by Zhongnan Hospital Ethics Committee (ethics code 2018025), and every patient signed informed consent.

### RNA Extraction and qRT-PCR

Total RNA was extracted using Trizol reagent (Invitrogen, United States), and cDNA was reverse transcribed from 1ug of purified RNA using PrimeScriptTM RT reagent kit (Toyobo, Osaka). The quantification of mRNA was examined using quantitative real-time polymerase chain reaction (qRT-PCR) on Biorad CFX (Biorad, United States), and gene expression was normalized by GAPDH. The primer sequences were obtained from PrimerBank and synthesized by TSINGKE (Wuhan, China) as follows: GAPDHF-5′CTGGGCTACACTGAGCACC3′, R-5′AAGTGGTCGTTGAGGGCAATG3′, SCG2F-5′ACCAGACCTCAGGTTGGAAAA3′, R-5′ACCAGACCTCAGGTTGGAAAA3′. We used the comparative CT (2-ΔΔCT) to calculate the gene mRNA expression levels, and the experiment was repeated three times with three biological replicates for each treatment.

### Protein Extraction and Western Blotting

According to the reagent instructions, total protein in tissues was extracted with RIPA lysis buffer (Boyotime, China), including a protease inhibitor cocktail (Thermo Scientific, United States). We performed western blot using standard methods with the specific antibody, SCG2 (1:1,000, Absin, abs117155), GAPDH (1:10000, Proteintech, 60004-1-Ig).

### Immunofluorescence

Tumor samples were immediately fixed with 4% Paraformaldehyde (PFA) for 1 h and dehydrated overnight at 4°C. We used the following primary antibodies for immunofluorescence: mouse anti-SCG2 (1:100, Absin, abs117155) and rat anti-CD68 (1:100, Novusbio, 100–683). Fluorescent secondary antibodies CY3-conjugated anti-mouse SCG2 (1:1,000) and FITC-conjugated anti-rat CD68 antibody (1:1,000) were incubated for 30 min at 25°C. Nuclei were counter stained with DAPI for 10 min. We randomly chose at least five optical fields (20× or 40× magnification) per tumor section for morphometric evaluation.

### Data Acquisition

RNA-sequencing expression data and clinicopathological information of 469 CRC patients were downloaded from The Cancer Genome Atlas (TCGA) database. GSE39582 cohort containing microarray gene expression profiles and clinical data was downloaded from the Gene Expression Omnibus (GEO) database. The proteome data of CRC patients was obtained from the Clinical Proteomic Tumor Analysis Consortium (CPTAC) Assay Portal (Whiteaker et al., 2014).

### SCG2 Expression Analysis

We identified SCG2 mRNA expression levels in multiple human cancers from the Tumor Immune Estimation Resource (TIMER) database (Li et al., 2017). In TCGA and GEO cohorts, the mRNA expression profiles of SCG2 were visualized, respectively. In the CPTAC cohort, the protein expression levels of SCG2 in 91 pairs of tumor and adjacent tissues were examined. qRT-PCR and WB were utilized to detect RNA and protein levels of SCG2. Finally, immunofluorescence assay was used to detect the expression and distribution of SCG2 protein in tumor and adjacent tissues of CRC patients.

### Survival Analysis and Significant Prognostic Marker Analysis

Patients were divided into high-SCG2 expression group and low-SCG2 expression group according to the median expression value of SCG2. We used R packages “survival” and “survminer” to analyze the correlation between SCG2 expression and overall survival (OS) time, disease-free survival (DFS) time. Besides, univariate and multivariate survival analyses were applied to examine the independent prognostic factors in CRC, using the “coxph” function in the “survival” package.

### SCG2 Related Genes and Functional Enrichment Analysis

To further study the SCG2-related molecular mechanisms, we performed differential expression genes (DEGs) analysis based on the medium value of SCG2 expression using the package “DESeq2.” Genes were ranked with positive correlation coefficients with SCG2 (Log2FoldChange ≥ 1, *p* < 0.01). Then Gene Ontology (GO) and Kyoto Encyclopedia of Genes and Genomes (KEGG) enrichment analysis were conducted using “enrichGo” and “enrichKEGG” functions in the “clusterprofiler” package. We conducted a PPI network based on DEGs by Cytoscape software, and its plug-in “ClueGO” was applied for further function enrichment analysis.

### Gene Set Enrichment Analysis

We performed Gene Set Enrichment Analysis (GSEA) to further understand the upregulated pathways in the high SCG2 expression group by GSEA 4.1 software. The number permutations for each gene were set to 1,000, false discovery rate (FDR) < 0.5 and normalized enrichment score (NES) > 1 were set at the cut-off criteria.

### Characterization of the TME

Tumor infiltrating immune cells (TIICs) in tumor samples and normal samples of the TCGA cohort were calculated using the CIBERSORT algorithm based on RNA-seq (Newman et al., 2015). The signature TIICs matrix “LM22” was set to 1,000 permutations. Tumor immune single-cell analysis was investigated with the “Dataset” module in Tumor Immune Single Cell Hub (TISCH, http://tisch.comp-genomics.org), which built a Single-cell RNA sequencing (scRNA-seq) atlas of 76 high-quality tumor datasets across 27 cancer types from GEO and ArrayExpress (Sun et al., 2021). Thirteen kinds of major lineage immune cells from GSE146771 cohort were analyzed.

### Correlation Analysis of SCG2 and TIICs

We applied the Estimation of STromal and Immune cells in MAlignant Tumours using Expression data (ESTIMATE) algorithm to calculate stromal and immune scores that predicted the levels of infiltrating stromal and immune cells in each patient (Yoshihara et al., 2013) and analyzed the correlations between SCG2 expression and the two scores. We further compared the proportions of twenty-two immune cell phenotypes in SCG2 high or low expression group using CIBERSORT algorithm. The correlations between SCG2 and tumor-associated macrophages (TAMs), M2 gene markers were analyzed to identify the relevance of SCG2 and macrophage polarization. The correlations between SCG2 expression and gene markers of various immune cells including CRC immune checkpoint genes were analyzed by TIMER (Li et al., 2017).

### Statistical Analysis

SPSS Statistics 26, GraphPad prism 8, R 4.0.2 were used for statistical analyses. The measurement data were presented as mean ± standard deviation. Statistical differences between two groups were examined by Wilcoxon signed rank test or Mann-Whitney U test. Statistical differences across three or more groups were examined by Kruskal-Wallis H test. Correlations were examined with the Spearman rank correlation test (weakly correlation when R > 0.1, moderate correlation when R > 0.3, strong correlation when R > 0.5). Statistically significant differences were considered when *p* < 0.05.

## Results

### Expression Profiles of SCG2 in Human Cancers

**FIGURE 1:**
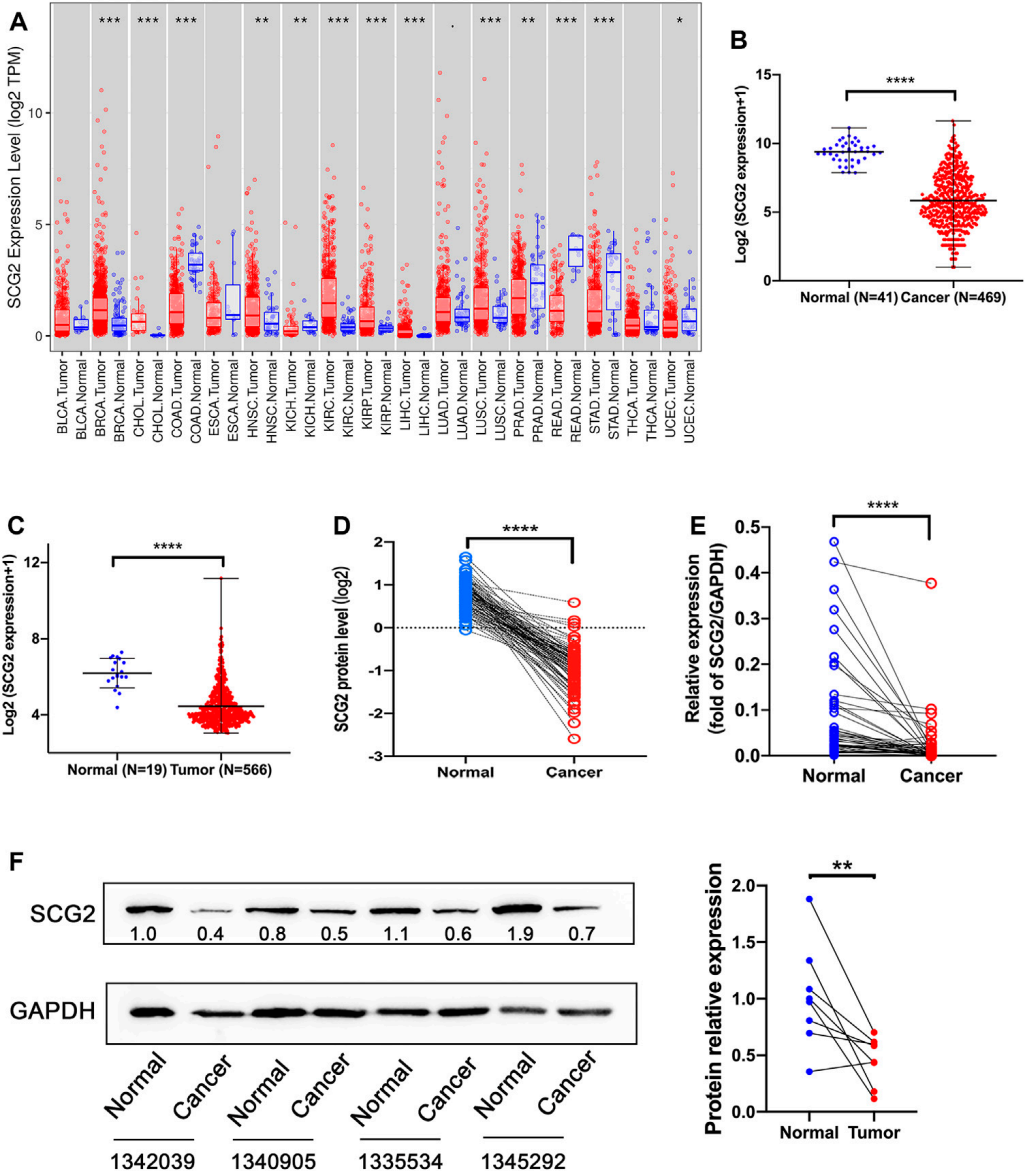
Expression analysis of SCG2. **(A)** The SCG2 expression level in multiple cancer types from TCGA database. **(B,C)** SCG2 mRNA expression levels in normal and tumor tissues of CRC from TCGA and GEO database. **(D)** SCG2 protein levels in 96 paired CRC tissues from CPTAC. **(E)** SCG2 mRNA expression level in tumor and adjacent samples. **(F)** Quantification of SCG2 protein expression in tumor and adjacent samples. **(A–C)** Examined by Mann-Whitney U test; **(D–F)** Examined by Wilcoxon signed rank test; *p < 0.05, **p < 0.01, ***p < 0.001, ****p < 0.0001.

To evaluate SCG2 expression in various human cancers, we analyzed TCGA RNA-seq using TIMER database. The SCG2 expression levels were higher in breast invasive carcinoma (BRCA), cholangiocarcinoma (CHOL), head and neck cancer (HNSC), kidney renal clear cell carcinoma (KIRC), kidney renal papillary cell carcinoma (KIRP), liver hepatocellular carcinoma (LIHC), lung squamous cell carcinoma (LUSC) compared with adjacent normal tissues. However, SCG2 expression was significantly lower in colon adenocarcinoma (COAD), kidney chromophobe (KICH), prostate adenocarcinoma (PRAD), rectum adenocarcinoma (READ), stomach adenocarcinoma (STAD), uterine corpus endometrial carcinoma (UCEC) compared with adjacent normal tissues (Figure 1A). These results suggested SCG2 expressed abnormally in various tumor types.

We then assessed SCG2 mRNA expression levels in TCGA and GEO cohorts and found significantly lower SCG2 expression levels in cancer tissues than normal tissues (Figures 1B,C). SCG2 protein expression was also lower in cancer tissues than adjacent tissues in CPTAC database (Figure 1D). To confirm the expression profile data, we examined mRNA and protein levels in tumor and normal tissues of CRC patients using qRT-PCR and Western blot (Figures 1E,F).

**FIGURE 2:**
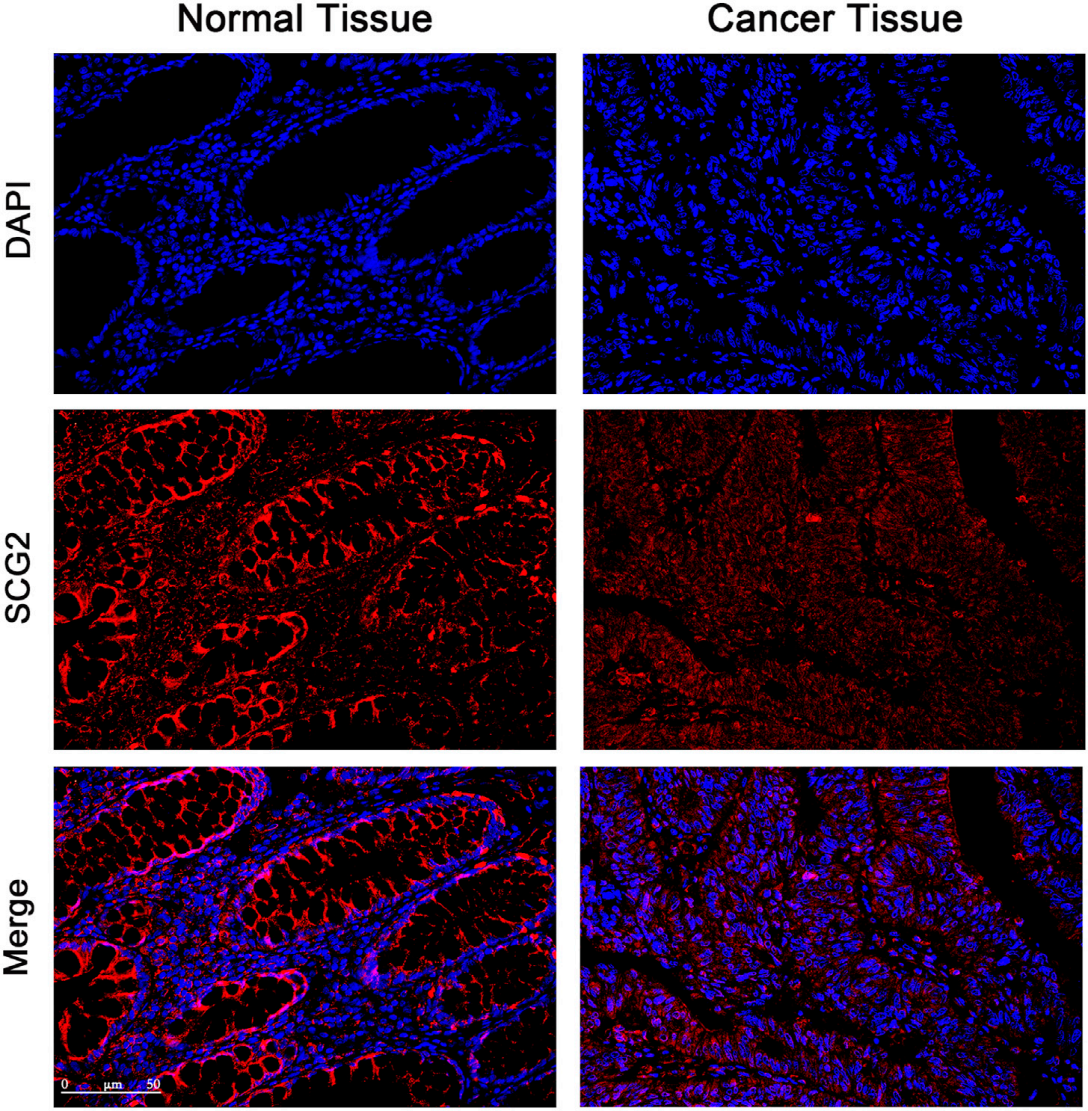
Immunofluorescence images of SCG2 (red) and DAPI (blue) in tumor and adjacent tissues of CRC patients. Seven pairs of tumors and adjacent tissues were examined, six of the tumor tissues showed deceased SCG2 expression compared to adjacent normal tissues.

The expression and distribution of SCG2 protein in tumor and adjacent tissues were monitored by immunofluorescence microscopy (Figure 2). In normal tissues, SCG2 was mainly distributed in colonic glands, possibly related to intestinal fluid secretion, whereas in tumor tissues, the intestinal mucosal tissues were destroyed and SCG2 expression decreased.

### Prognostic Significance of SCG2 Expression in CRC

**FIGURE 3:**
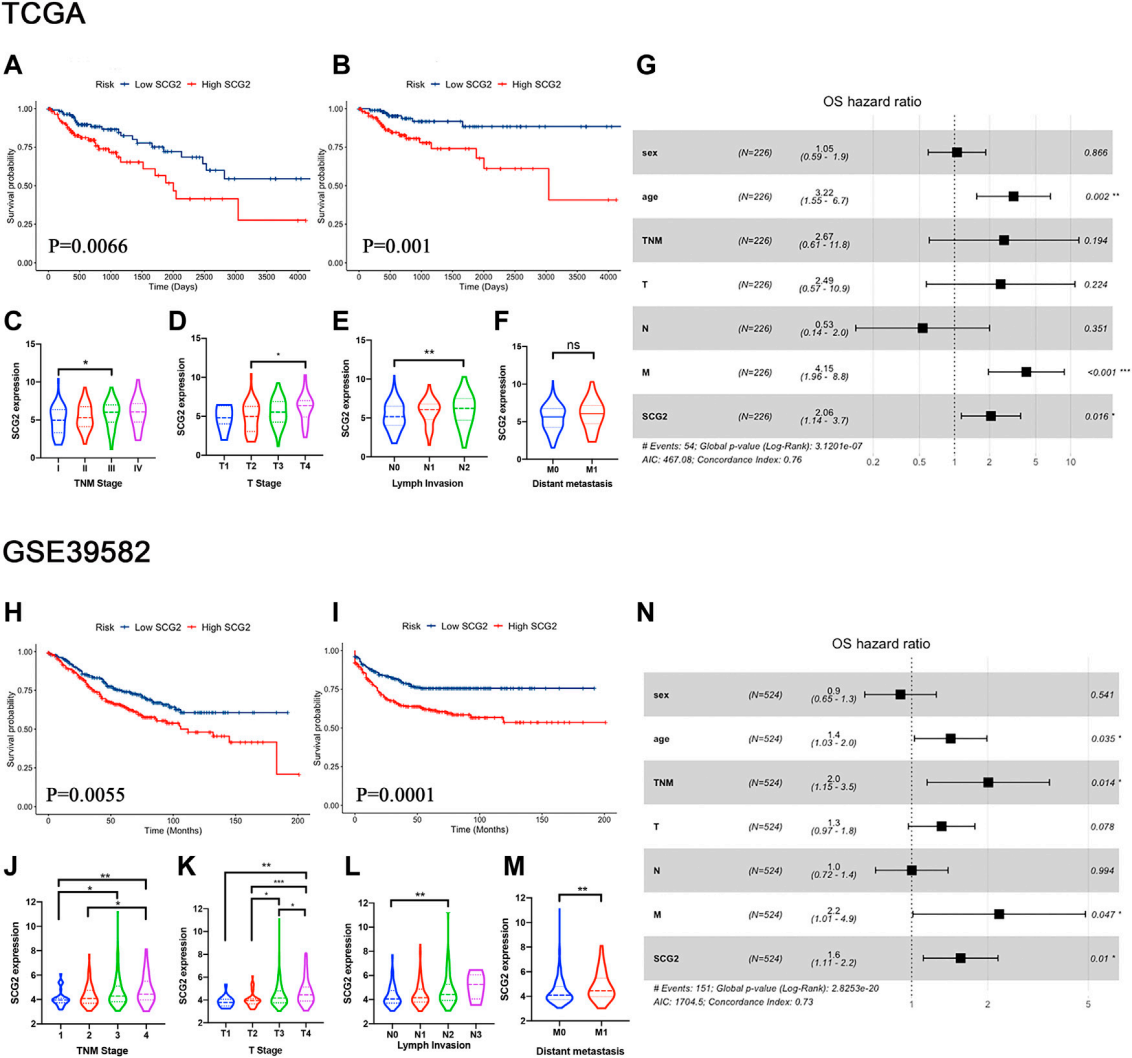
High SCG2 expression is associated with worse survival and advanced clinical stage in CRC patients. **(A,B)** OS, DFS in the TCGA CRC cohort. **(C–F)** SCG2 expression was higher in patients of advanced T, N, M, TNM stage in the TCGA CRC cohort. **(G)** Multivariate Cox analysis of patients in the TCGA CRC cohort showing the HRs of different factors. **(H,I)** OS, DFS in the GSE39582 CRC cohort. **(J–M)** SCG2 expression was higher in patients of advanced T, N, M, TNM stage in the GSE39582 CRC cohort. **(N)** Multivariate Cox analysis of patients in the GSE39582 CRC cohort showing the HRs of different factors. HRs, hazard ratios. **(C–E,J–L)** Examined by Kruskal-Wallis H test; **(F,M)** Examined by Mann-Whitney U test; *p < 0.05, **p < 0.01, ***p < 0.001.

A univariate Cox survival analysis based on TCGA database indicated that TNM stage (*p* = 3.6e-04), Invasion depth (*p* = 0.031), Lymph node metastasis (*p* = 0.002), distant metastasis (*p* = 4.96e-07), and SCG2 expression (*p* = 0.008) were significant risk factors for overall survival (Table 1). Survival analysis using the Kaplan-Meier method showed that higher SCG2 expression was significantly correlated with shorter OS (*p* = 0.009) and DFS (*p* < 0.001) in TCGA cohorts (Figures 3A,B). SCG2 mRNA expression was associated with advanced TNM stage, T stage, and lymph invasion (Figures 3C–F). The forest plot based on multivariate Cox regression analysis showed that age (*p* = 0.002), M stage (*p* = 2.07e-04), and SCG2 expression (*p* = 0.016) were independent prognosis factors of CRC patients (Figure 3G). The above results were verified in independent cohort GSE39582 (Table 1; Figures 3H–N). Besides, we used the patients from our hospital to analyze the correlation between SCG2 expression and tumor histological grading, there was no significant difference (Supplementary Figure S1A). Overall, elevated SCG2 mRNA expression was significantly associated with poor prognosis and advanced clinicopathological parameters in CRC patients.

**TABLE 1:** Univariate Cox regression of prognostic factors in CRC patients.

Parameters	TCGA	Univariate cox regression		GSE39582	Univariate cox regression	
	HR (95%CI)	z-score	*p*-value	HR (95%CI)	z-score	*p*-value
Sex (female vs. male)	0.809 (0.469–1.395)	-0.761	0.446	1.127 (0.823–1.542)	0.744	0.457
Age (≥60 vs. <60)	1.781 (0.918–3.456)	1.707	0.088	1.318 (0.9675–1.796)	1.751	0.080
TNM stage (III-IV vs. I-II)	2.739 (1.575–4.764)	3.568	3.6e-04	2.337 (1.871–2.918)	0.113	7e-14
Invasion depth (T3/T4 vs. T1/T2)	4.748 (1.152–19.57)	2.155	0.031	1.948 (1.478–2.567)	4.372	2.23e-06
Lymph node metastasis (N1/N2/N3 vs. N0)	2.308 (1.345–3.962)	3.036	0.002	1.479 (1.239–1.764)	4.339	1.43e-05
Distant metastasis (M1 vs. M0)	4.379 (2.462–7.788)	5.028	4.96e-07	7.779 (5.360–11.29)	10.79	2e-16
SCG2 expression (High vs. low)	2.111 (1.127–3.659)	2.661	0.008	1.510 (1.111–2.053)	2.629	0.009

HR: hazard ratio; CI: confidence interval.

### Analysis of SCG2 Co-expressed Genes

Genes in co-expression modules are always involved in same biological pathways (Stuart et al., 2003) and have disease predictive value (Yang et al., 2014). We performed genetic difference analysis based on SCG2 medium expression level. The heat map displayed differential expressed genes according to the Pearson correlation (Supplementary Figure S1B). We verified the correlations between SCG2 and MYH11, SYNPO2, DDR2, FABP4, TNS1 in TIMER (all R > 0.5, *p* < 0.0001, shown in Supplementary Figures S1C–G). Furthermore, high expressions of FABP4, DDR2, and TNS1 were associated with worse overall survival in CRC patients (Supplementary Figures S1H–J), MYH11 and TNS1 were associated with advanced clinical stage (Supplementary Figures S1K,L). It was possible that abnormal expression of SCG2 and these genes involved in CRC progression contributing to a worse prognosis.

To explore the potential molecular function of SCG2 in CRC, we performed KEGG pathway enrichment and GO analyses with the SCG2 co-expressed genes. KEGG enrichment analysis showed that these genes were associated with many signaling pathways (Supplementary Table S1), including known cancer-related pathways such as the PI3K-Akt signaling pathway and ECM-receptor interaction pathway. GO annotations further suggested these genes were associated with many biological processes (Supplementary Table S2)

We then performed PPI network analysis based on SCG2 co-expressed genes in TCGA and GSE39582 cohort (Supplementary Figure S2A). 56 seed and cluster genes in the network were selected for further enrichment analysis (Supplementary Figure S2B). The result indicated that SCG2 and its co-expressed genes could directly or indirectly regulate various immune-related processes, including macrophage polarization (Supplementary Figure S2C).

### GSEA Validates SCG2-Related Pathways

To further study the SCG2-associated signaling pathways in CRC, GSEA was performed between samples in low and high SCG2 expression groups. GSEA identified a total of 20 considerably upregulated hallmark pathways in the high SCG2 expression group (Supplementary Table S3). The tumor related pathways involved in tumorigenesis, tumor invasion, and metastasis included “Kras signaling up,” “Hedgehog signaling,” “Notch signaling,” “Wnt beta catenin signaling,” “TGF beta signaling,” “Epithelial mesenchymal transition,” and “Angiogenesis.” The inflammatory and immune related pathways included “Complement,” “IL2 STAT5 signaling,” “Inflammatory response,” “IL6 JAK STAT3 signaling,” and “Allograft rejection.” A summary of the enrichment results is shown in Supplementary Figure S3.

### Evaluation of TIICs in CRC

**FIGURE 4:**
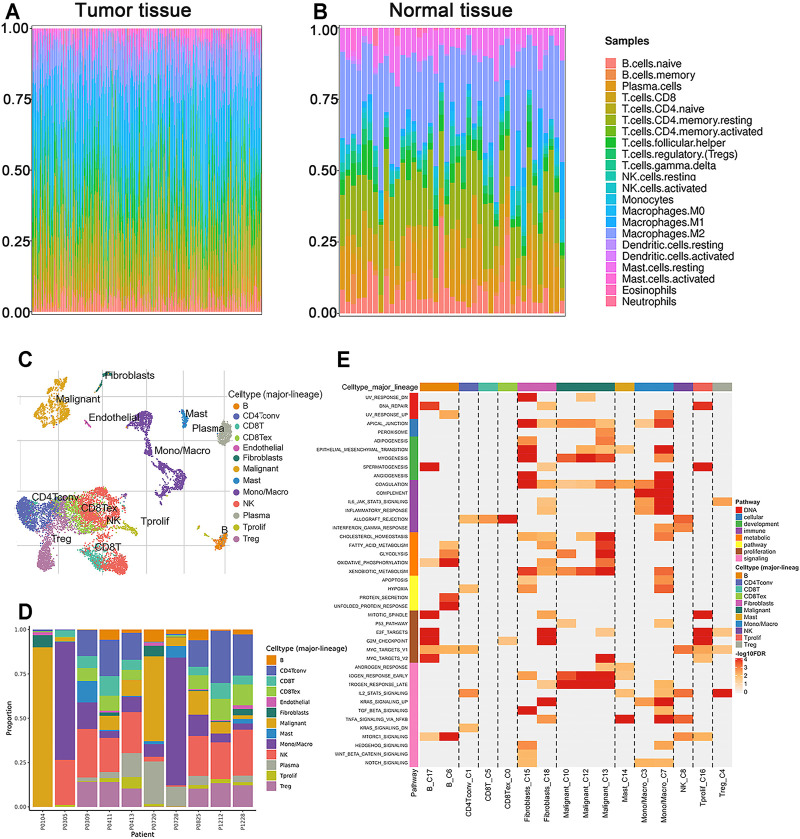
The proportions of TIICs in TME and enrichment analysis by GSEA. **(A,B)** Composition of 22 immune cells in the tumor and normal tissues of CRC patients in TCGA cohort. **(C,D)** Components of TME at the single-cell resolution of CRC patients in GSE146771 cohort. **(E)** Enriched upregulated hallmark pathways in different immune cells.

TIIC is an integral part of tumor immune microenvironment (Deschoolmeester et al., 2010). In the current study, we estimated the proportion of twenty-two immune cells in the tumor and normal samples of TCGA using the CIBERSORT algorithm (Figures 4A,B). The composition of immune cells in TME varied significantly between both intragroup and intergroup. Additionally, the components of TME at single-cell resolution were investigated using TISCH database. TIICs of GSE146771 cohort and each patient’s cell type proportion were displayed (Figures 4C,D). GSEA showed the enriched upregulated hallmark pathways in different cells (Figure 4E). Interestingly, the pathways enriched in monocyte-macrophages, “Epithelial mesenchymal transition,” “Angiogenesis,” “Complement,” “Inflammatory response” and “IL6 JAK STAT3 signaling,” were also significantly enriched in high SCG2 expression group (Supplementary Figure S3).

### SCG2 Expression Significantly Correlate With TIICs in CRC

**FIGURE 5:**
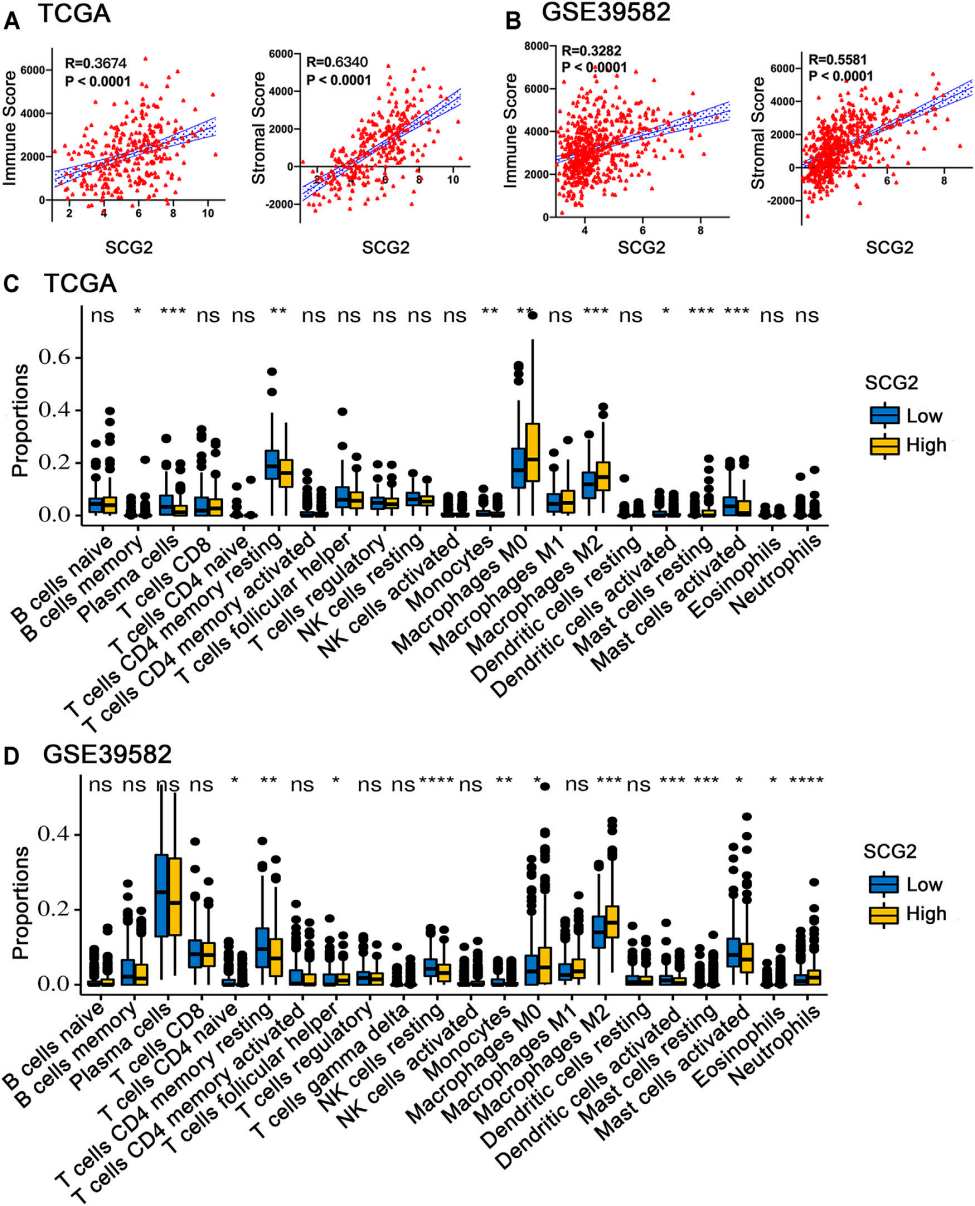
Correlation between SCG2 expression and TIICs. **(A)** The correlation analysis between SCG2 expression and immune score, stromal score in TCGA cohort. **(B)** The correlation analysis between SCG2 expression and immune score, stromal score in GSE39582 cohort. **(C,D)** Twenty-two kinds of tumor-infiltrating immune cells are plotted according to the SCG2 expression level in TCGA and GSE39582 cohort. **(A,B)** Examined by Spearman’s correlation analysis; **(C,D)** Examined by Mann-Whitney U test; *p < 0.05, **p < 0.01, ***p < 0.001, ****p < 0.0001.

We conducted ESTIMATE analysis to explore the relationship between SCG2 and TME. The results suggested that high SCG2 expression was significantly associated with higher immune score and stromal store in CRC (Figures 5A,B). CIBERSORT analysis showed that the proportions of resting memory CD4 cells, monocytes, activated dendritic cells, and activated mast cells were downregulated in the high SCG2 expression group, whereas M0 macrophages, M2 macrophages, and resting mast cells were significantly upregulated in the high SCG2 group (Figures 5C,D). We further performed correlation analysis between SCG2 expression and various TIICs gene markers (Table 2). Significant correlations were observed between SCG2 expression and T cell exhaustion (immune checkpoint genes), general T cells, CD8 + T cells, CD4 + T cells, Th1, Tfh, Th17, Monocyte, M1 macrophages, M2 macrophages, natural killer (NK) cell, dendritic cells, neutrophils in CRC. The results demonstrated that SCG2 expression was correlated with TME and TIICs in CRC.

**TABLE 2:** Correlation analysis between SCG2 and markers of immune cells.

Terms	Markers	None		Purity	
		Cor	*p*	Cor	*p*
T cell exhaustion	PDCD1 (PD-1)	0.226	***	0.244	***
	CTLA4	0.265	***	0.277	***
	CD274 (PD-L1)	0.280	***	0.289	***
	PDCD1LG2 (PD-L2)	0.379	***	0.376	***
	HAVCR2	0.435	***	0.36	***
	TIGIT	0.338	***	0.351	***
	BTLA	0.253	***	0.253	***
	CD244	0.091	0.052	0.104	0.036
	CD96	0.245	***	0.254	***
	IDO1	0.208	***	0.221	***
	KDR	0.509	***	0.521	***
	TGFBR1	0.554	***	0.545	***
	GZMB	0.045	0.341	0.062	0.215
	LAG3	0.206	***	0.232	***
Monocyte	CD86 (B7-2)	0.438	***	0.435	***
	CSF1R	0.426	***	0.418	***
TAM	CCL2	0.510	***	0.495	***
	CD68	0.340	***	0.348	***
	IL10	0.295	***	0.312	***
M1 Macrophage	IRF5	0.251	***	0.258	***
	INOS (NOS2)	0.222	***	0.189	**
	COX2 (PTGS2)	0.176	**	0.172	**
M2 Macrophage	CD163	0.467	***	0.465	***
	VSIG4	0.432	***	0.419	***
	MS4A4A	0.415	***	0.498	***
CD8+T	CD8A	0.227	***	0.240	***
	CD8B	0.135	*	0.139	*
CD4+T	CD4	0.398	***	0.396	***
	CD40LG (CD40L)	0.147	*	0.154	*
	CXCR4	0.432	***	0.436	***
T cell (general)	CD3D	0.124	*	0.133	*
	CD3E	0.230	***	0.252	***
	CD2	0.219	***	0.232	***
	CD28	0.350	***	0.356	***
Th1	TBX21	0.239	***	0.270	***
	STAT4	0.228	***	0.228	***
	STAT1	0.305	***	0.325	***
	IFNG	0.074	0.114	0.082	0.100
Th2	STAT6	0.141	*	0.142	*
	STAT5A	0.131	*	0.151	*
Th17	STAT3	0.278	***	0.293	***
	IL17A	0.238	***	0.239	***
Treg	FOXP3	0.311	***	0.321	***
	STAT5B	0.341	***	0.365	***
	TGFB1	0.473	***	0.472	***
	CD25 (IL2RA)	0.289	***	0.290	***
B cell	CD19	0.207	***	0.221	***
	CD79A	0.272	***	0.284	***
Neutrophils	CD66b (CEACAM8)	−0.271	***	−0.283	***
	CD11b (ITGAM)	0.423	***	0.427	***
	CCR7	0.243	***	0.264	***
Natural Killer cell	CD16 (FCGR3A)	0.476	***	0.473	***
	CD56 (NCAM1)	0.448	***	0.447	***
	KIR2DL1	0.052	0.266	0.063	0.206
	KIR2DL3	0.076	0.106	0.094	0.058
	KIR2DL4	0.069	0.139	0.084	0.090
	KIR3DL1	0.109	0.020	0.125	0.012
	KIR3DL2	0.111	0.018	0.131	*
Dendritic cell	HLA-DRA	0.267	***	0.266	***
	HLA-DPA1	0.328	***	0.324	***
	BOCA-1 (CD1C)	0.306	***	0.298	***
	BOCA-4 (NRP1)	0.605	***	0.612	***
	CD11c (ITGAX)	0.431	***	0.438	***

TAM, tumor-associated macrophage; Th, T helper cell; Treg, regulatory T cell.

None, correlation without adjustment; Purity, correlation adjusted by tumor purity.

Cor, R value of Spearman’s correlation. *p < 0.01, **p < 0.001, ***p < 0.0001.

### SCG2 Expression Associated With M2 Macrophage Polarization

**FIGURE 6:**
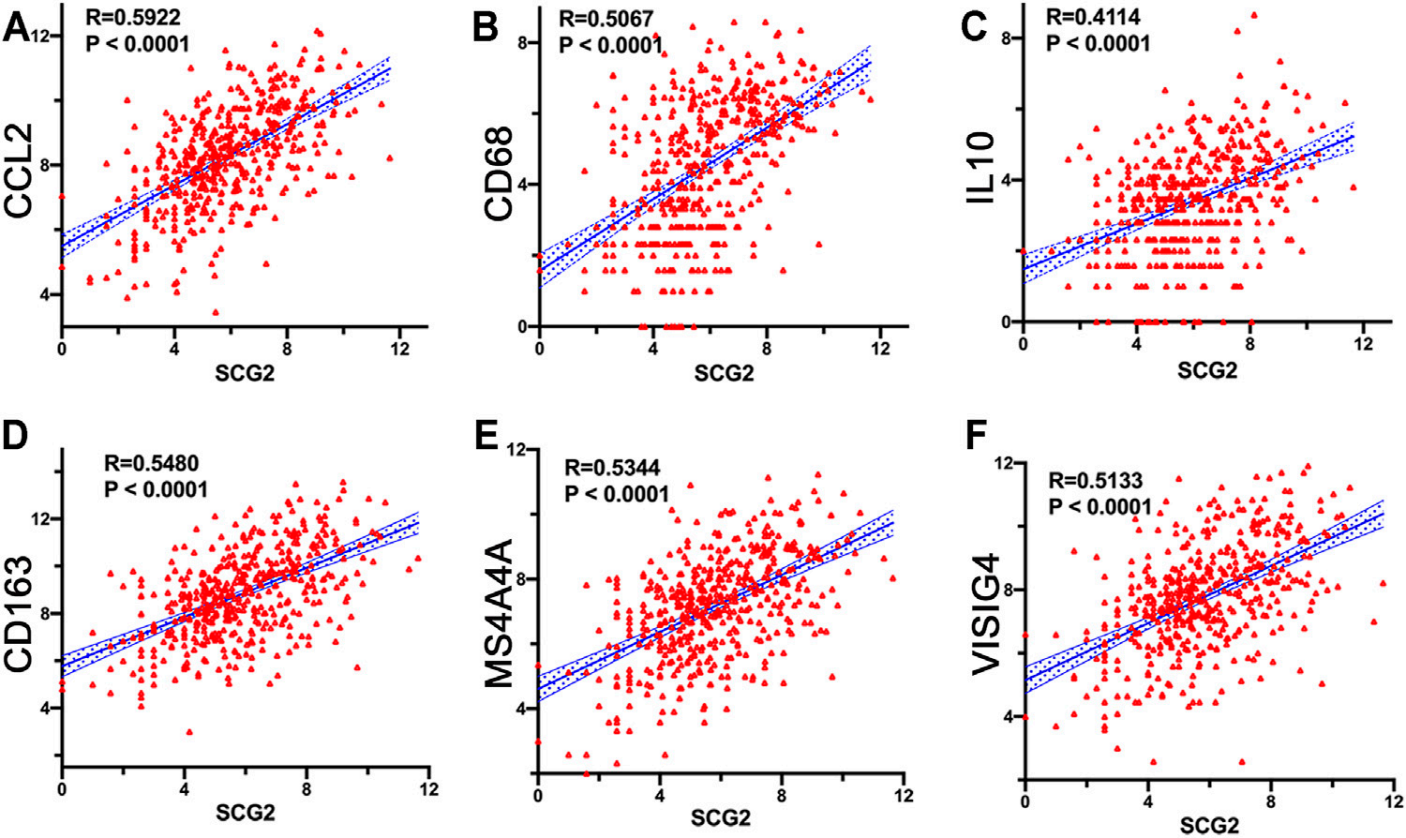
Correlation of SCG2 expression with macrophage polarization. **(A–C)** Correlation analysis between SCG2 expression and TAM markers genes (CCL2, CD68, IL10). **(D–F)** Correlation analysis between SCG2 expression and M2 Macrophage markers genes (CD163, MS4A4A, VSIG4). Examined by Spearman’s correlation analysis.

**FIGURE 7:**
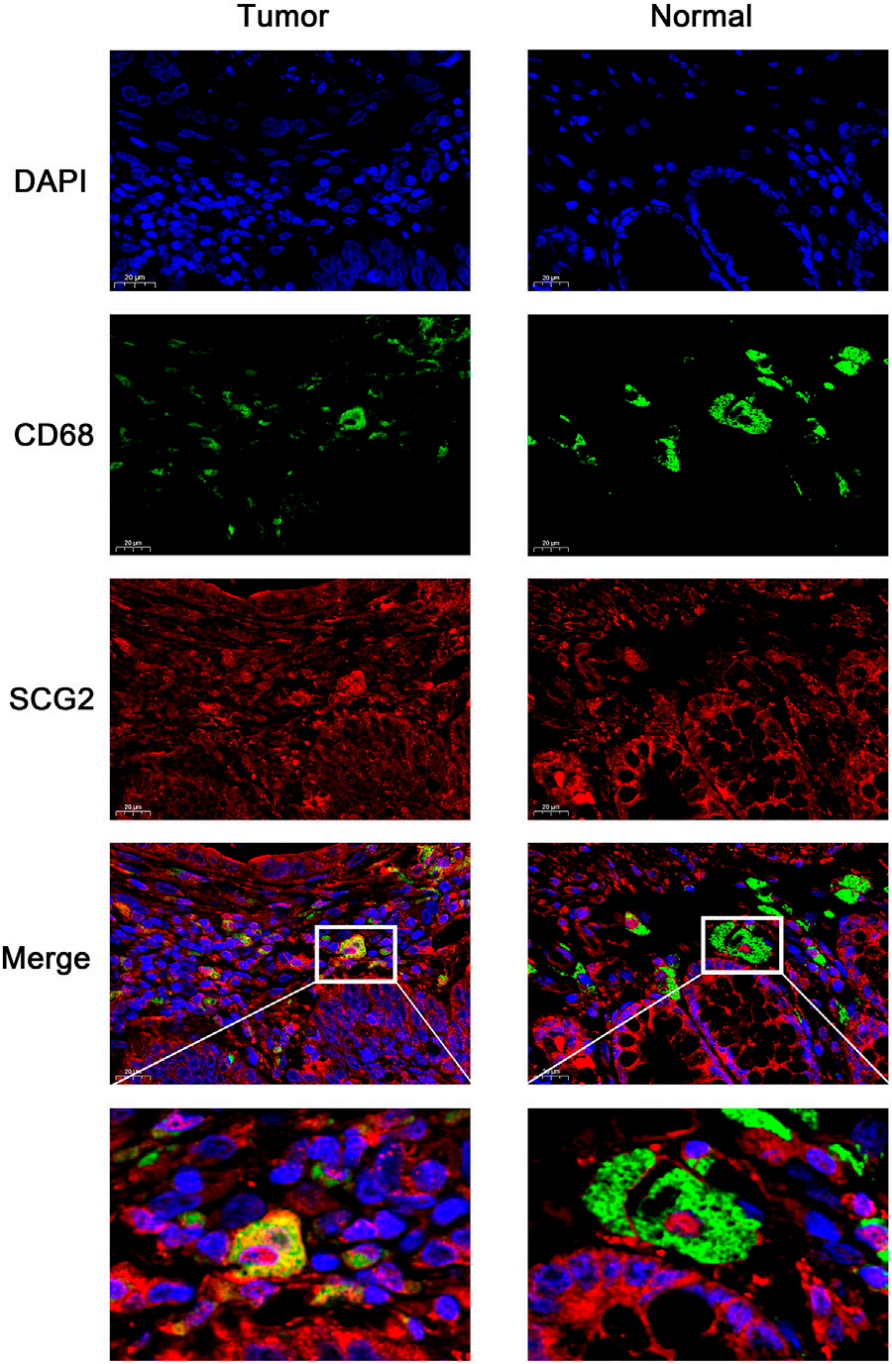
Immunofluorescence images of DAPI (blue), CD68 (green), SCG2 (red), and the merge photos in normal tissues and cancer tissues of CRC patients.

The expressions of TAM and M2 macrophage marker genes were higher in monocyte-macrophage cells from tumor samples than from normal samples or peripheral blood mononuclear cell (PBMC) (Supplementary Figure S4). It indicated that, in the TME, macrophages tend to polarize towards M2 phenotype and differentiate into TAMs thus performed pro-tumoral functions. CIBERSORT analysis suggested the proportion of M2 macrophages increased in the SCG2 high expression group, but the proportion of M1 macrophages not significantly changed (Figures 5C,D). Additionally, clear correlations existed between SCG2 expression and the marker genes of M2 macrophages and TAMs (Figures 6A–F). The aforementioned results prompted us to hypothesize that SCG2 could promote the polarization of M2 macrophages. To validate this conjecture, we performed confocal immunofluorescence microscopy to confirm the colocalization between SCG2 and macrophage. As shown in Figure 7, SCG2 was colocalized with macrophages in tumor tissues, whereas there was no apparent colocalization in normal tissues. Altogether, the above results suggested that SCG2 might promote tumor infiltrating macrophages polarization toward M2 phenotype and play a pro-tumoral role in CRC.

## Discussion

CRC is a common health problem and one of the leading causes of cancer death worldwide (Ferlay et al., 2015). Despite numerous studies effort to improve our surgical treatment, radiotherapy, chemotherapy, and immunotherapy over the years, the prognosis of patients with advanced CRC remains poor (Seymour et al., 2007). In the present study, we explored the role of SCG2 in colorectal cancer, revealed its prognostic value, biological functions, associated pathways, and regulation of tumor immunity by analyzing open-access databases comprehensively.

SCG2 expression was remarkably decreased in tumor tissues compared with normal tissues of CRC. In normal tissues, our results showed that SCG2 was mainly distributed in colonic glands, which possibly related to intestinal fluid secretion. In contrast, in tumor tissues, the intestinal mucosal tissues were destroyed and total SCG2 expression was decreased. Additionally, SCG2 expression was associated with T, N, M, and TNM stages in CRC patients. Higher expression of SCG2 was significantly correlated to worse OS and DFS. Multivariate cox analysis also showed that SCG2 expression level was an independent prognostic marker in CRC.

To investigate the biological roles of SCG2, we carried out a gene differential expression analysis based on SCG2 median expression level. Then functional enrichment analyses were performed using the genes positively correlated with SCG2. The results suggested that SCG2 was strongly correlated with MYH11, SYNPO2, DDR2, FABP4, and TNS1, which were all involved in tumorigenesis and inflammatory immune response (Gan et al., 2019; Gao et al., 2020; Liu et al., 2020; Matrai et al., 2020; Sun et al., 2020; Tian et al., 2020). Higher expression of these genes might associate with worse survival and higher clinical stage of CRC patients.

Functional enrichment analysis revealed that SCG2 was correlated with several tumorigenesis related pathways such as “PI3K-Akt,” “Wnt,” and “TGF-beta pathways” (Schatoff et al., 2017; Koveitypour et al., 2019). A few pathways related to tumor metastasis included “ECM-receptor interaction” and “focal adhesion” (Gu et al., 2018; Machackova et al., 2020). Furthermore, numerous genes were involved in immune-related processes. “Regulation of actin cytoskeleton” plays a vital role in immune response, tumor migration, and invasion (Schmitz et al., 2000; C. Wickramarachchi et al., 2010); “B cell receptor” is initiated via interaction between B cell receptor and specific antigens and plays a crucial role in immune system (Li et al., 2019); “leukocyte transendothelial migration” tightly regulates inflammation and is associated with the transient crossing of leukocytes through the blood vessel wall (Schimmel et al., 2017). These results indicated that SCG2 might have regulatory roles in tumor progression and immune moderation.

The result of GSEA suggested high SCG2 expression might activate the IL6 JAK STAT3 signaling pathway and IL2–STAT5 signaling pathway in CRC patients. Previous studies have shown that STAT3 was activated in TIICs, including TAMs, amplifying immune suppression, and targeting IL-6 to regulate STAT3 signaling pathway was considered a potential immunotherapy for CRC (Wang and Sun, 2014; Verdeil et al., 2019). STAT5 also played a critical role in tumor immunity, regulated Treg cell’s function and development. Consistent activation of STAT5 was associated with suppressing antitumor immunity and increased tumor proliferation and invasion (Rani and Murphy, 2016). These findings indicated SCG2 might relate to the efficiency of immunotherapy in CRC patients.

The TIICs in TME are critical players in tumor progression, modulate tumor inflammation and metastasis variously (Gonzalez et al., 2018). We quantified each CRC sample’s immune and stromal cell infiltration levels using immune and stromal scores evaluated by ESTIMATE algorithm, respectively. Our results showed the two scores were significantly correlated with SCG2 expression in CRC patients. Some studies have demonstrated that both immune and stromal scores were associated with poor prognosis in CRC patients (Liu et al., 2020; Yuan et al., 2020).

CIBERSORT analysis showed that multiple immune cells had significantly different proportions between SCG2 high and low expression groups. Notably, the proportions of M0 and M2 macrophages in the SCG2 high expression group were considerably higher than those in the SCG2 low expression group, whereas M1 macrophages showed no significant change. Besides, SCG2 expression was moderately or strongly correlated with M2 macrophage marker genes and TAM marker genes but was slightly correlated with M1 macrophage marker genes. Similarly, confocal immunofluorescence microscopy showed SCG2 had an apparent colocalization with macrophages in tumor tissues. The previous study has demonstrated that SN, SCG2 derived peptide, could induce macrophage accumulation and angiogenesis (Liu et al., 2018). These results indicated that high expression of SCG2 might promote M0 macrophages polarize to M2 and eventually differentiate into TAMs, enhancing tumor cell invasion, metastasis, angiogenesis, and inhibiting the anti-tumoral immune surveillance (Kim and Bae, 2016), predict a worse prognosis of CRC patients (Waniczek et al., 2017).

We also found a positive correlation between SCG2 expression and immune checkpoint markers PD-1, PD-L1/2, CTLA-4, TIM-3, TIGIT in CRC patients. The binding of PD-L1/2 to the PD-1 receptor leads to T-cell function’s deactivation, allowing tumor cells to evade immune attacks (Chen and Han, 2015). Upregulation of PD-1 and Tim-3 was associated with the poor prognosis of CRC patients in stage I-III (Kuai et al., 2020), and high expression of TIGIT was associated with advanced TNM stage and poor DFS in CRC patients with mismatch repair deficiency (Zhou et al., 2020). The correlations between SCG2 expression and immune checkpoint genes indicated a potential mechanism of SCG2 regulation on T cell exhaustion and provided a new target for tumor immunotherapy.

However, our study had some limitations. First, we conducted analysis mainly based on samples from TCGA and GEO cohorts, it would be better to confirm the findings in a larger sample size. Second, genetic difference analysis based on RNA-seq might not be precise. Additionally, the mechanisms of SCG2 regulating the infiltration of immune cells and macrophage polarization require further experimental investigation, which would be a future direction for our research.

In conclusion, our study revealed that overexpression of SCG2 predicted poor prognosis and advanced clinical stage in CRC patients. SCG2 was associated with tumor immune cells infiltration, promoted M2 macrophage polarization, and correlated with immune checkpoint expression in CRC. In summary, SCG2 played a critical role in the regulation of tumor immunity and made it a potential biomarker and therapeutic target in CRC.

## Data Availability

The datasets presented in this study can be found in online repositories. The names of the repository/repositories and accession number(s) can be found in the article/Supplementary Material.
