# Supplementary material for: SCG2 is a Prognostic Biomarker Associated With Immune Infiltration and Macrophage Polarization in Colorectal Cancer
Source: Front Cell Dev Biol. 2022 Jan 3;9:795133. doi: 10.3389/fcell.2021.795133 (PMC8763391; doi:10.3389/fcell.2021.795133)
Supplement: Supplementary file 3 [file Image4.pdf]

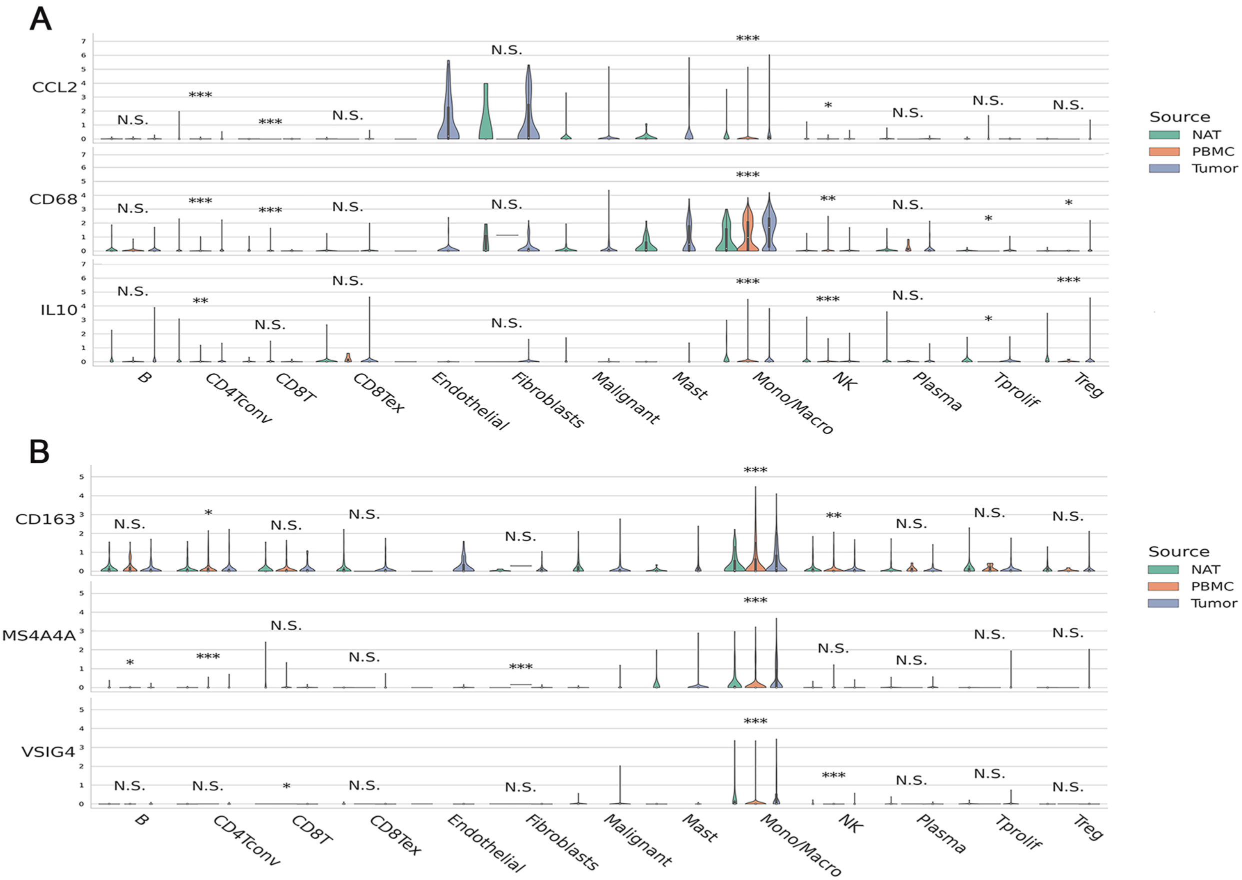

Supplementary Figure 4. (A)TAM marker genes (CCL2, CD68, IL10) and (B) M2 macrophage marker genes (CD163, MS4A4A, VSIG4) expression levels in normal tissue, PBMC, and tumor samples of CRC patients. Kruskal-Wallis test:N.S 0.05<q<=1, * 0.01<q<=0.05, **0.001<q<=0.01, ***q<=0.001.
PBMC, peripheral blood mononuclear cell.
