# Supplementary material for: SCG2 is a Prognostic Biomarker Associated With Immune Infiltration and Macrophage Polarization in Colorectal Cancer
Source: Front Cell Dev Biol. 2022 Jan 3;9:795133. doi: 10.3389/fcell.2021.795133 (PMC8763391; doi:10.3389/fcell.2021.795133)
Supplement: Supplementary file 4 [file Image2.PDF]

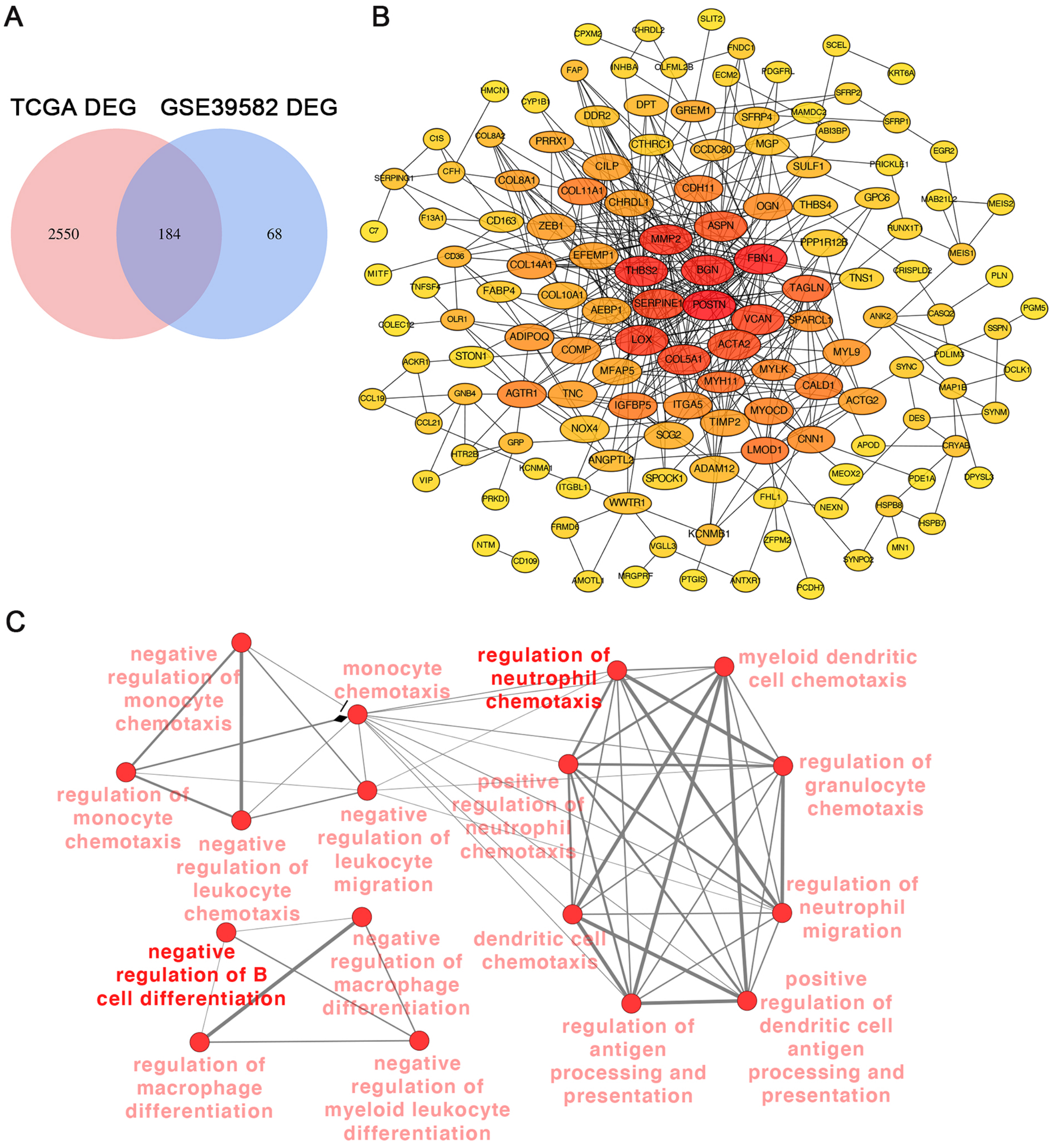

Supplementary Figure 2. (A) SCG2 co-expressed genes in TCGA and GSE39582 cohort. (B) PPI network of SCG2 co-expressed genes. (C) SCG2 related immune processes.
