# Supplementary material for: SCG2 is a Prognostic Biomarker Associated With Immune Infiltration and Macrophage Polarization in Colorectal Cancer
Source: Front Cell Dev Biol. 2022 Jan 3;9:795133. doi: 10.3389/fcell.2021.795133 (PMC8763391; doi:10.3389/fcell.2021.795133)
Supplement: Supplementary file 5 [file Image3.PDF]

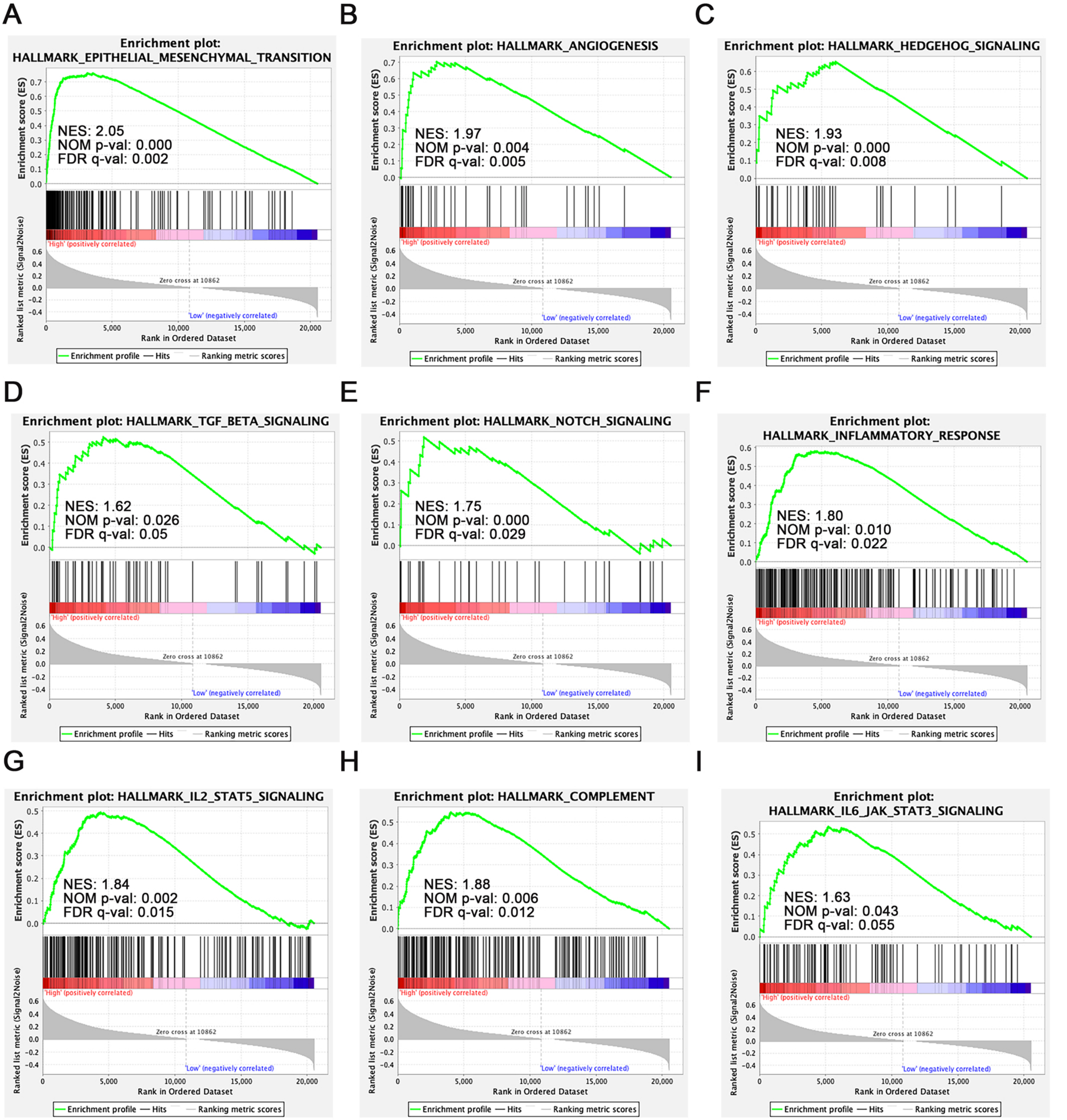

Supplementary Figure 3. Pathway enrichment analysis by GSEA. (A-E) Five cancer-promoting signaling pathways enriched in the SCG2 high-expression group, including epithelial mesenchymal transition, angiogenesis, Hedgehog signaling, TGF beta signaling, and Notch signaling. (F-I) Four immune-related pathways included Inflammatory response, IL2 STAT5 signaling, complement, IL6 JAK STAT3 signaling.
