# Supplementary material for: SCG2 is a Prognostic Biomarker Associated With Immune Infiltration and Macrophage Polarization in Colorectal Cancer
Source: Front Cell Dev Biol. 2022 Jan 3;9:795133. doi: 10.3389/fcell.2021.795133 (PMC8763391; doi:10.3389/fcell.2021.795133)
Supplement: Supplementary file 7 [file Image1.PDF]

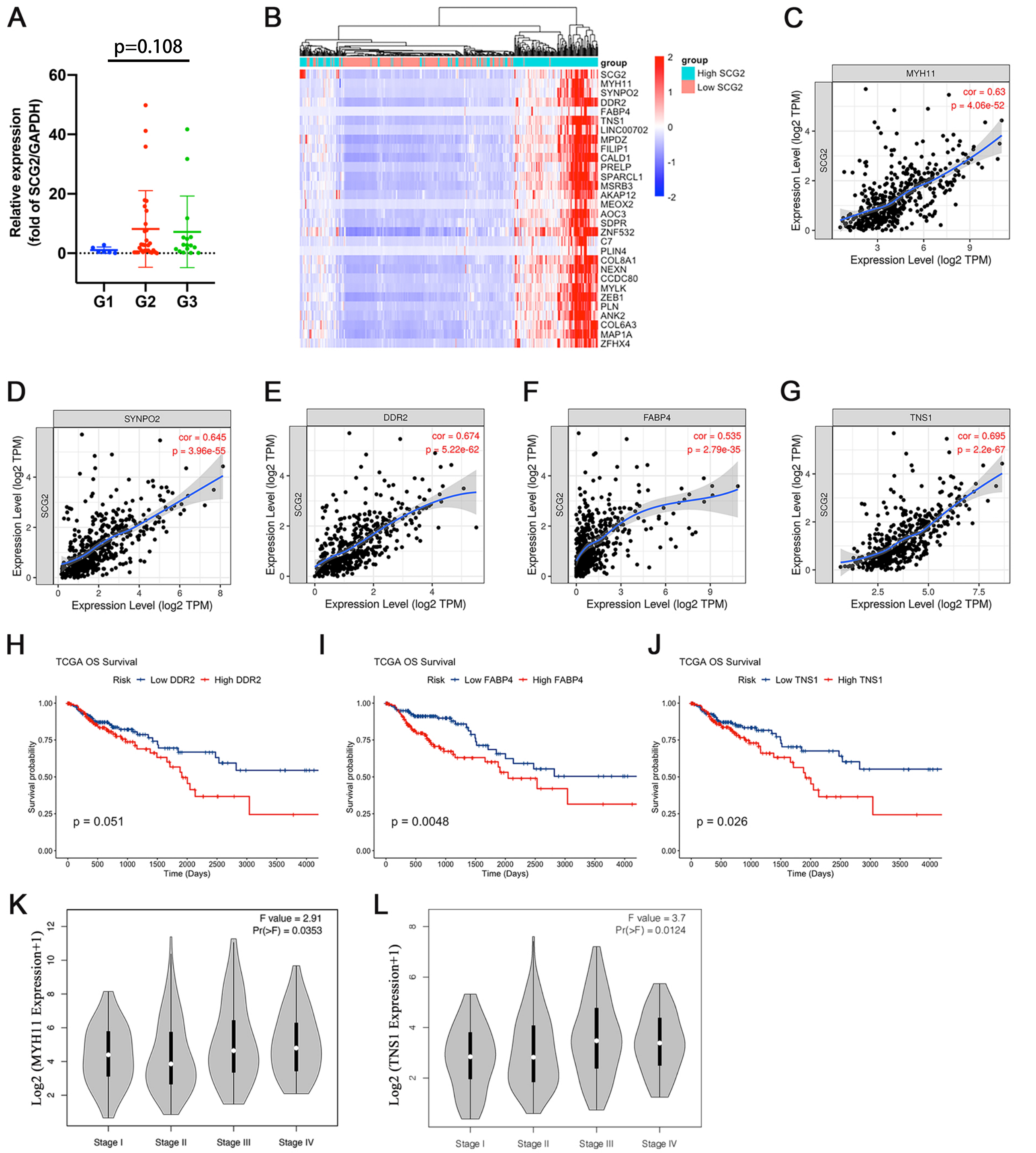

Supplementary Figure 1. (A) SCG2 expression levels in different tumor grades. (B) Heatmap of SCG2 related genes. (C-G) Correlation analysis between SCG2 and top 5 positive related genes MYH11, SYNPO2, DDR2, FABP4, TNS1. (H-J) Overall Survival curves of DDR2, FABP4, TNS1 high and low expression from TCGA database. (K,L) MYH11 and TNS1 expression levels in different stages of CRC from GEPIA database.
